# Seroprevalence and Risk Factors of Cystic Echinococcosis in Cattle and Buffaloes: Insights From an In‐House ELISA

**DOI:** 10.1002/vms3.70901

**Published:** 2026-03-20

**Authors:** Mughees Aizaz Alvi, Talha Javaid, Majed H. Wakid, Hong‐Bin Yan, Muhammad Wasim Usmani, Li Li, Warda Qamar, Aliza Ali, Junaid Amjed, Wan‐Zhong Jia, Muhammad Saqib

**Affiliations:** ^1^ State Key Laboratory of Animal Disease Control and Prevention, College of Veterinary Medicine Lanzhou University, Lanzhou Veterinary Research Institute, Chinese Academy of Agricultural Sciences Lanzhou China; ^2^ National Para‐Reference Laboratory for Animal Echinococcosis, Gansu Province Research Center for Basic Disciplines of Pathogen Biology Key Laboratory of Veterinary Parasitology of Gansu Province, Key Laboratory of Veterinary Etiological Biology and Key Laboratory of Ruminant Disease Prevention and Control (West), Ministry of Agricultural and Rural Affairs Lanzhou China; ^3^ Department of Clinical Medicine and Surgery University of Agriculture Faisalabad Pakistan; ^4^ Department of Medical Laboratory Sciences, Faculty of Applied Medical Sciences King Abdulaziz University Jeddah Saudi Arabia; ^5^ Special Infectious Agents Unit, King Fahd Medical Research Center King Abdulaziz University Jeddah Saudi Arabia; ^6^ Institute of Drug Discovery Technology Ningbo University Ningbo Zhejiang China; ^7^ Department of Parasitology University of Agriculture Faisalabad Pakistan

**Keywords:** *Echinococcus granulosus*, ELISA, livestock, One Health, Pakistan, seroprevalence

## Abstract

**Background:**

Cystic echinococcosis (CE) is a zoonotic disease caused by the larval stage of *Echinococcus granulosus* and associated with productivity and economic losses in the livestock population across the world.

**Objectives:**

This cross‐sectional study spanned over a period of 1 year (2024) to determine the seroprevalence and associated risk factors of CE in large ruminants (cattle and buffaloes) hosted in different districts of Punjab, Pakistan, using an in‐house developed ELISA.

**Methods:**

Overall, 430 serum samples from 197 buffaloes and 233 cattle were collected for detection of anti‐*E. granulosus* antibodies using an in‐house ELISA.

**Results:**

The highest seropositivity rate (68.24%) was in cattle, whereas buffaloes had 19.28%. The geographical variation revealed that the lowest seropositivity rate (9.52%) was in Haroonabad, whereas Bahawalpur had the highest seropositivity rate (96.29%). Sex‐wise, females showed a seroprevalence of 46.13%, whereas males had a seroprevalence of 37.50%. Age‐wise trends showed that seropositivity increased up to 10–12 years of age, while decreasing in older animals. Breed‐wise, the highest seropositivity rate of 96.29% was observed in the Cholistani cattle, and the lowest rate of 25.25% was shown by Nili‐Ravi buffaloes. The LASSO model revealed a high prediction accuracy of 76.98% and an area under the curve (0.73), suggesting geographical and host‐related impacts on the epidemiology of CE. However, our findings indicate widespread seropositivity, the study does not evaluate production losses or economic impact, and our conclusion refers to epidemiological patterns.

**Conclusions:**

There is a prompt need for targeted strategies under the One Health concept for region‐specific surveillance, improving animal husbandry practices and controlling definitive hosts, particularly in high‐risk areas of Pakistan.

## Graphical abstract

This study reveals widespread exposure to *Echinococcus granulosus* in cattle and buffaloes across Punjab, Pakistan (overall seroprevalence 45.81%), with marked geographic, species, age, sex and breed‐related differences. Findings highlight high‐risk areas and support targeted, One Health–based surveillance and control strategies.

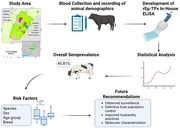

## 1 Introduction

Cystic echinococcosis (CE), a zoonotic global disease which is transmitted by the larval stage of *Echinococcus granulosus*, has caused a negative impact on the livestock productivity and poses a serious health risk, especially in regions where the animal husbandry practices encourage the successful completion of *E. granulosus* life cycle (Wen et al. 2019; Shoulah et al. 2023). To complete the life cycle of *E. granulosus*, the parasite requires two different categories of hosts: final hosts (canids) and intermediate hosts (ungulates). Individuals who accidentally consume *E. granulosus* eggs may develop liver and lung cysts (Thompson 2017). Because the disease decreases meat and milk production and causes the condemnation of contaminated offal, CE also leads to economic losses (Budke et al. 2006).

ELISA tests are one of the serological diagnostic methods that are used to detect anti‐*E. granulosus* antibodies in animals with varying sensitivity and specificity (Craig et al. 2007). Large‐scale animal screening and the early detection of anti‐*E. granulosus* antibodies prior to the development of clinical signs and visible cysts are facilitated by ELISAs (Siracusano et al. 2012; Toaleb et al. 2023). It is noteworthy to mention that ELISA‐based seropositivity indicates exposure to *E. granulosus* antigens and does not always reflect the active cystic disease. The serology of circulating IgG antibodies can be retained after previous exposure or at an early stage of infection; hence, the serology results should be considered a manifestation of the exposure and not as the active cyst formation and related loss of production.

Seroprevalence studies conducted in various countries of the Asian continent show that CE is endemic in the livestock population. For example, seroprevalence rate of CE in cattle raised in Xinjiang province of China ranges from 15% to 20%, whereas 17.3% seroprevalence has been recorded in yaks and cattle reared in Qinghai‐Tibet area of China (Tian et al. 2018; Wang et al. 2023). There are more than 47.7 million heads of buffalo and 59.7 million heads of cattle in Pakistan (Pakistan Economic Survey 2024). Being a neglected zoonotic disease, there is scarcity of CE seroprevalence data in the livestock population of Pakistan (Alvi et al. 2020b).

This study was planned to determine the seroprevalence and to identify the risk factors of CE in large ruminant populations in the Punjab province of Pakistan by using an in‐house developed ELISA. The results of this study provide valuable insights into the epidemiology of CE and identify associated risk factors.

## 2 Materials and Methods

### 2.1 Study Areas, Sampling Frame and Sample Size

**FIGURE 1:**
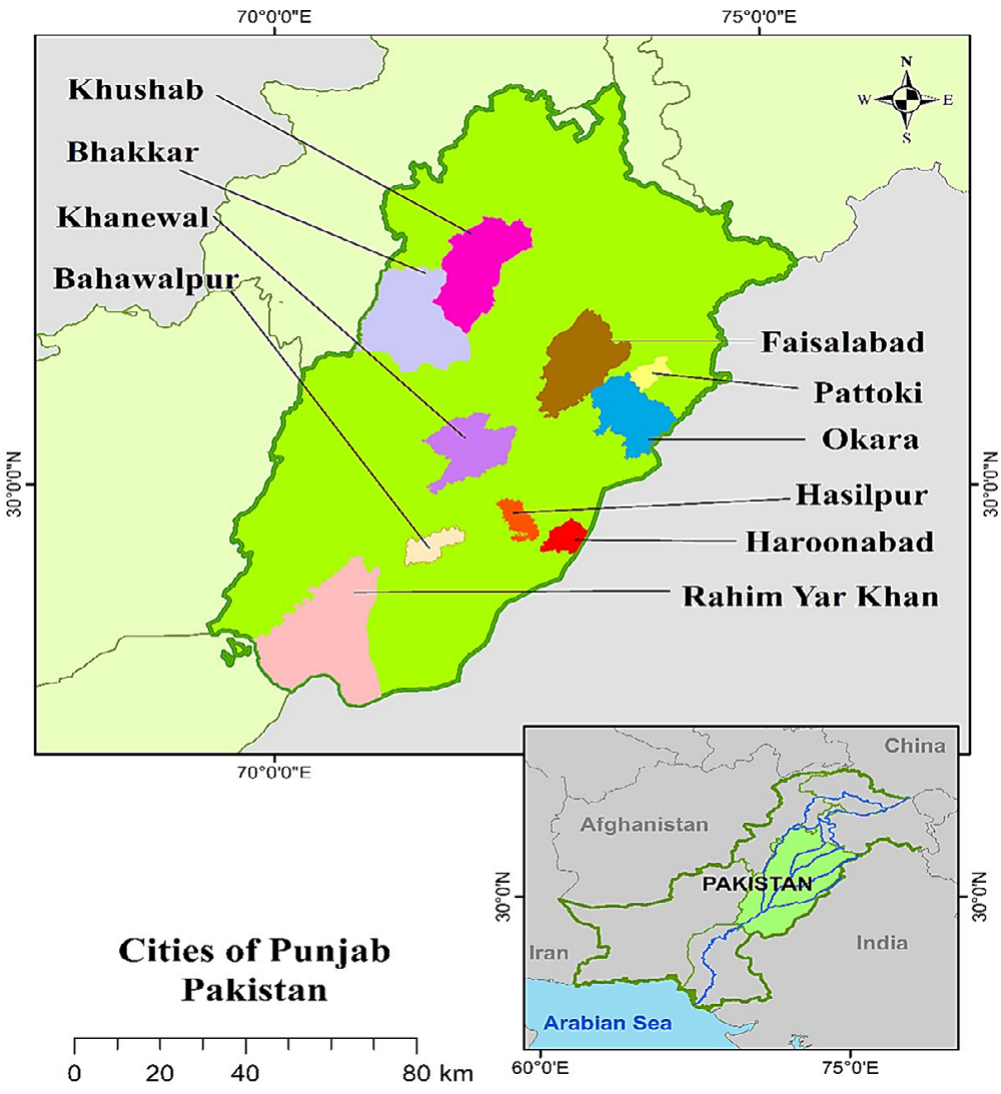
Map of Pakistan showing different study areas.

Cross‐sectional sero‐epidemiological research between January and December 2024 was executed in ten cities in the Punjab province of Pakistan, including Faisalabad, Okara, Pattoki, Bahawalpur, Bhakkar, Khanewal, Khushab, Haroonabad, Hasilpur and Rahim Yar Khan (Figure 1).

The selection of the study locales was done in a purposive manner and was aimed at representing various geographic areas and established patterns of livestock migration and population density. Our sampling approach of choice in each area was stratified convenience sampling: Serum samples were opportunistically collected among animals found at (i) the commercial and smallholder farms and (ii) local slaughterhouses. In farms, the animals were chosen by chance out of agreeing owners so that the sampled animals were representative of any available age or breed; at slaughterhouses, the sampled animals were those presented to the slaughterhouse on a sampling day when owner/abattoir assent was given. The requirements to include were bovine species (cattle or buffalo), the availability of the sample during field trips and the consent of the owner of the sample or the slaughterhouse to collect the blood. Animals were not included when samples were severely hemolyzed and when acute illness rendered the safe collection of blood impossible. Blood samples were collected in gel clot serum vials, properly labelled and sent for serological investigation to the laboratory of Veterinary Preventive Medicine and Public Health, Department of Clinical Medicine and Surgery, University of Agriculture, Faisalabad, Pakistan. The serum samples were maintained at −20°C after being separated by centrifugation at 3000 rpm for 10 min in gel clot vials.

The desired sample size was calculated using the CE prevalence figures in Pakistan. The frequency of CE in cattle and buffalo populations has previously been surveyed in various areas, and it has been indicated that the range is normally between 5% and 18%. The conventional formula (*n* = *Z* ^2^ *p*(1 − *p*)/*d* ^2^) was used to compute the required minimum sample size, where *Z* ^2^ = 12.57, *p* = 0.15, which was calculated as the mean of these literature values, and *d* = 0.10 in order to achieve an absolute precision of 5% at 95% confidence. The result was *n* ≈ 196. We also calculated a lower bound requirement, as there is a spatial variation in CE prevalence, and the variance of a fraction is maximized when *p* = 0.50. When *p* = 0.50 and *d* = 0.05 are used, a maximum sample size of *n* = 384 is required. A total of 430 animals were tested, including cattle (*n* = 233) and buffaloes (*n *= 197). The sample size of 430 animals achieved thus surpasses the criteria used in the literature (*n* = 196) as well as the conservative estimate (*n* ≈ 384), which is fit adequately to determine the prevalence of overall CE. The data relevant to animal demographics, including species, breed, sex and age, were collected after visual examination of animal and owner interviews. Seven groups of age groups were categorized (≤4, 4–6, 6–8, 8–10, 10–12, 12–14 and >14 years). Sahiwal, Cholistani, Nili‐Ravi and crossbreds were among the selected breeds.

### 2.2 ELISA Development and Testing

Cysts from recently killed cattle were used to extract hydatid cystic fluid (HCF) aseptically. *E. granulosus* was naturally present in all of the animals. Necropsy‐based identification of cysts followed by the *cox*1 gene amplification was the primary reference method for confirmation of *E. granulosus* cysts.

Although the germinal layer of cysts was utilized for the infertile cysts, the DNA was taken from the protoscoleces of the viable cysts. To sediment the protoscoleces, the HCF from the viable cysts was centrifuged at 2000 × *g* for 20 min at 4°C. Sterilized falcon tubes were used to collect the clear supernatant HCF, which was then kept at −70°C until it was needed.

Recombinant antigen of *E. granulosus* thioredoxin peroxidase (rEg‐TPx) was prepared as described (Liang et al. 2020) and used for Bovine Echinococcosis ELISA test development. Briefly, rEg‐TPx was diluted in 0.1 M carbonate buffer (pH 9.6) at a concentration of 1 µg/mL and incubated overnight at 4°C in 96‐well polystyrene plates, 100 µL per well. After washing with PBS (pH 7.4) and 0.05% Tween‐20 three times for 5 min each wash, the plates were coated with 5% casein diluted in PBS (blocking buffer) at 37°C for 2 h. Serum samples were diluted 1:100 times in PBS, then added to the plate (100 µL per well) and incubated at 37°C for 30 min. After the plates were washed as described above, HRP‐labelled goat whole molecule anti‐cow IgG H&L (Abcam, ab6934; diluted 1:40,000 in PBS) was added to the plates (100 µL/well) at 37°C for 30 min. Finally, the reaction was developed in the dark with 100 µL/well of 3,3,5,5 tetramethylbenzidine (Surmodics, TMBW‐1000‐01) for 15 min and stopped by the addition of 100 µL 2 M H_2_SO_4_. The optical density (OD) of the wells was determined at 450 nm using an iMark Microplate reader (BIO‐RAD).

Animals infected with *E. granulosus* and animals grown in helminth‐free control circumstances yielded positive and negative control sera, respectively. The absorbance readings of the negative control sera plus three times the standard deviation were used to determine the cut‐off value. As (i) the absorbance value of the substrate blank was less than 0.20 and (ii) the ratio of the mean values of the positive and negative controls was greater than samples, all tests were deemed valid. A total of 500 negative serum samples from healthy bovines were tested in the optimal conditions, and the cut‐off value was calculated as the mean OD450 plus three standard deviations of the OD450.

The cut‐off values were used as a standard for subsequent test results. Positive predictive value: Mean OD sample ≥ cut‐off value. Negative predictive value: Mean OD sample/P < cut‐off value. Both the intra‐ and inter‐assay variabilities were <10%.

Sensitivity of the assay was detected using 100 serum samples from *E. granulosus*‐infected bovines. The analytical sensitivity of rEg‐TPx was 82.4%. The analytical sensitivity percentage was calculated as [ELISA positive/(True positive + False negative)] × 100.

Specificity was determined using 50 serum samples from bovines infected with other pathogens: brucellosis (*n* = 10), *Taenia multiceps* (*n* = 20), babesiosis (*n* = 10) and bovine skin nodular disease (*n* = 10). The analytical specificity percentage was calculated as [ELISA negative/(True negative + False positive)] × 100. The analytical specificity determined using 50 serum samples from bovines infected with other pathogens was 97.5% for rEg‐TPx.

### 2.3 Statistical Analysis

Descriptive statistics were used to compute the overall and stratified seroprevalence by district, species, breed, age and sex. The Chi‐square test was used to evaluate the relationships between seropositivity and categorical variables. The study used binary logistic regression to find out the significant variables of seropositivity. Calculations were used to determine 95% confidence intervals (CIs) and odds ratios (ORs). Every variable's impact on seropositivity was evaluated through marginal effects analysis. To have adjusted estimates and considering all the possible confounders, all the factors (species, sex, age group, breed and city) were entered into the multivariate logistic regression model at the same time. The stabilised coefficients and the prevention of overfitting due to the sparse categories and multicollinearity were achieved by penalised logistic regression with L1 regularisation (LASSO). Regional trends in seropositivity were identified through clustering analysis (*K*‐means algorithm). Area under the curve (AUC) values and receiver operating characteristic (ROC) curve analysis were used to evaluate the prediction ability of logistic models.

## 3 Results

The overall seroprevalence of 45.81% (197/430) had a good precision of 95% CI of 41.10%–50.50% which is enough considering the target sample size. The statistical power of this comparison (>99) was very high in view of the fact that seroprevalence in cattle and buffalo differed significantly. Table 1 shows the prevalence rates and 95% CI in different categories.

**TABLE 1:** Seroprevalence of anti‐*Echinococcus granulosus* antibodies in cattle and buffaloes in Punjab, Pakistan.

Category	Variable	Positive/Tested	Prevalence (95% CI)
District	Bahawalpur	26/27	96.29 (81.72–99.34)
	Rahim Yar Khan	12/16	75.00 (50.50–89.82)
	Bhakkar	60/92	65.21 (50.06–74.16)
	Pattoki	22/41	53.65 (38.75–67.95)
	Hasilpur	8/19	42.10 (23.15–63.73)
	Okara	28/68	41.17 (30.26–53.04)
	Khushab	19/56	33.92 (22.92–47.00)
	Faisalabad	10/38	26.31 (14.97–42.01)
	Khanewal	10/52	19.23 (10.80–31.90)
	Haroonabad	2/21	9.52 (2.65–28.91)
Species	Cattle	159/233	68.24 (62.01–73.88)
	Buffalo	38/197	19.28 (14.39–25.37)
Sex	Female	191/414	46.13 (41.40–50.96)
	Male	6/16	37.50 (18.48–61.36)
Age (Years)	6–8	33/56	58.92 (45.88–70.83)
	4–6	6/11	54.54 (28.01–78.73)
	10–12	42/78	53.84 (42.87–64.47)
	8–10	61/126	48.41 (39.86–57.05)
	>14	17/36	47.22 (31.98–62.99)
	12–14	19/47	40.42 (27.64–54.66)
	<4	19/76	25.00 (16.63–35.78)
Breed	Cholistani	26/27	96.29 (81.72–99.34)
	Sahiwal	118/193	61.13 (54.11–67.74)
	Cross‐bred	4/13	30.76 (12.68–57.63)
	Nili‐Ravi	49/197	25.25 (19.35–31.35)

Abbreviation: CI, confidence intervals.

Bahawalpur showed the highest seropositivity (96.29%; 26/27). *E. granulosus* Bhakkar provided the most samples (*n* = 92), and 65.22% (60/92) of them tested positive. In Faisalabad, 10/38 animals (26.32%) were found seropositive. The seroprevalence (9.52%; 2/21) was lowest in Haroonabad. In Hasilpur, the seropositivity rate was 42.10% (8/19). Moreover, other cities had different seropositivity levels: Okara had a moderate seropositivity rate of 41.17% (28/68), Pattoki reported 53.65% (22/41) and Khushab showed a seropositivity rate of 33.92% (19/56). The lowest seroprevalence rate was 19.23% (10/52) found in Khanewal. These findings show that cattle and buffaloes in the tested different districts of Punjab, Pakistan, have significantly different geographic exposures to *E. granulosus*.

The results of the Chi‐square test for independence among cities and seropositivity were statistically significant, with a *p* value <0.0001, Chi‐square statistic of 142.40 and degrees of freedom 9. This shows that the seroprevalence of animals testing positive for *E. granulosus* antibodies is strongly correlated with the city of origin.

Seropositivity was employed as a result, and the city was the predictor in a logistic regression. A probable outcome of seropositivity varied statistically significantly across a number of cities, showing that infection risk varies by geography. Significant correlations between the city of origin and the odds of testing positive for *E. granulosus* antibodies were found by the logistic regression analysis. With a regression coefficient of −2.63 (*p* = 0.0116), animals from Bhakkar had considerably lower probability of seropositivity than those from the reference city. As demonstrated by a highly significant coefficient of −4.29 (*p* < 0.001), animals from Faisalabad also had a much‐reduced likelihood of testing positively. Haroonabad had the strongest negative relationship, with a coefficient value of −5.51 (*p* < 0.001), suggesting much reduced probabilities of infection in animals from this region. The odds of seropositivity showed a statistically significant decline in Hasilpur, with a coefficient value of −3.58 (*p* = 0.0014). These findings demonstrate the effect of geographic location on the seroprevalence of *E. granulosus* and confirm the existence of regional heterogeneity in exposure risk.

**FIGURE 2:**
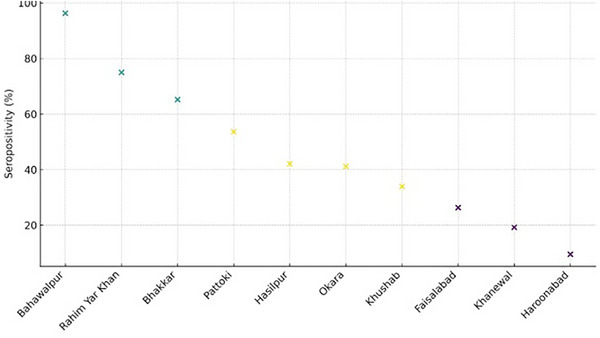
City‐wise clustering based on seropositivity. Each city is represented by its positive rate and colour‐coded according to its assigned cluster.

With *K*‐means analysis of *E. granulosus* seropositivity rates, three different groups were observed based on the city‐based clustering (Figure 2). Bahawalpur, Rahim Yar Khan and Bhakkar were categorized in Cluster 1, which included the high‐seropositivity group. Pattoki, Hasilpur, Okara and Khushab were grouped under Cluster 2, which was moderate seropositivity. Faisalabad, Khanewal and Haroonabad were classified as Cluster 3, which is the low‐seropositivity group. Such clusters indicate the existence of evident geographical disparities in *E. granulosus* exposure and the importance of regionally specific control strategies that would be structured in accordance with regional epidemiological trends.

**FIGURE 3:**
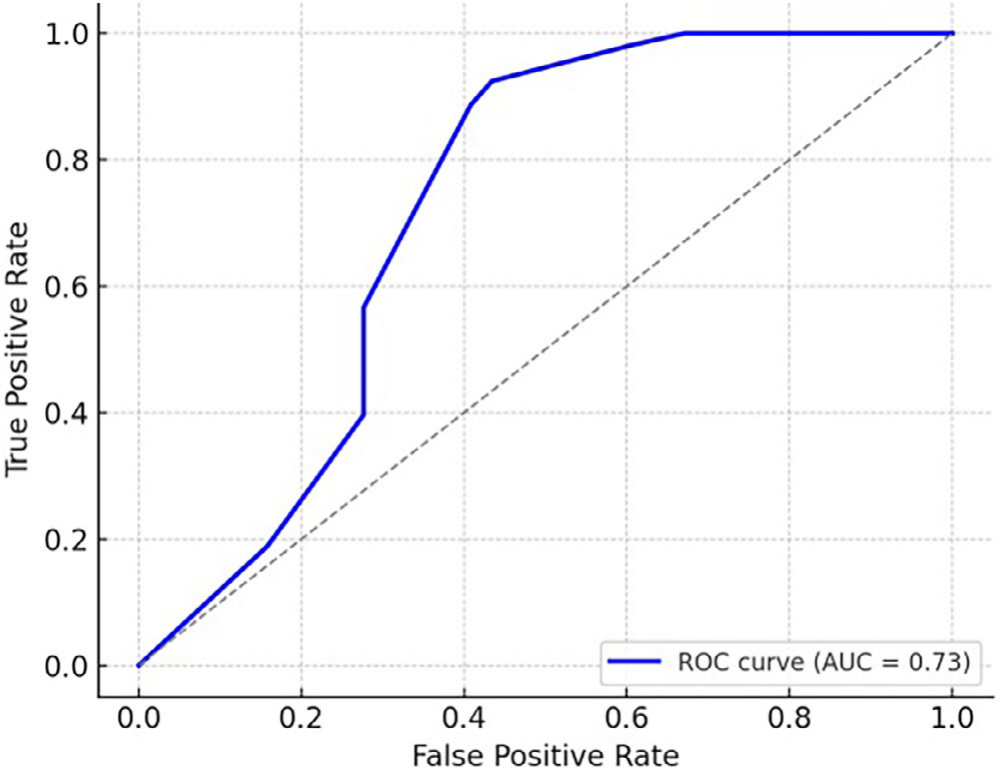
Receiver operating characteristic (ROC) curve for the city‐based logistic regression model used to predict seropositivity. AUC, area under the curve.

An AUC of 0.73 was obtained from ROC analysis of the logistic regression model that used city as a categorical predictor (Figure 3). This shows that, depending on the animal's city of origin, the model has a moderate predictive capacity to differentiate between seropositive and seronegative animals. Geographic location offers significant predictive value for seropositivity of *E. granulosus*, as seen by the ROC curve's excellent separation.

In the present investigation, a total of 430 blood samples collected from buffaloes (*n* = 197) and cattle (*n* = 233) were examined to find anti‐*E. granulosus* antibodies. Seroprevalence in buffaloes was 19.28% (38/197); however, the seropositivity rate was significantly greater in cattle (68.24%; 159/233). These results suggest that among the studied animal population, cattle had a much higher prevalence of *E. granulosus* antibodies than buffaloes.

The statistical significance of the association between seropositivity to *E. granulosus* and host species (cattle vs. buffalo) was analysed using a chi‐square test. The findings showed a strong significant association (*χ* ^2^ = 66.06, df = 1, *p* < 0.0001). According to the null hypothesis, the observed seropositivity rates in the cattle population were significantly greater than predicted, but the seropositivity rates in buffaloes were significantly lower than predicted. This shows that the probability of an animal being seropositive for *E. granulosus* is statistically significantly impacted by its species, with cattle exhibiting a significantly greater prevalence than buffaloes.

A binary logistic regression analysis was carried out using species as the predictor variable and seropositivity (positive/negative) as the outcome variable in order to further examine the association between animal species and seropositivity to *E. granulosus*. The model revealed that species had a statistically significant impact on the probability of a positive test result.

Odd ratios (OR = 5.87; *p* < 0.0001) revealed that cattle are almost six times more vulnerable to being seropositive as compared to buffaloes. These results support the chi‐square result and indicate a statistically significant association between host species and seropositivity, with cattle being markedly susceptible to *E. granulosus* than buffaloes.

Comparing cattle to buffaloes, marginal effects investigations show that cattle had a 35.9% (*dy*/*dx* = 0.359) higher likelihood of testing positive for *E. granulosus* antibodies. This effect has a 95% CI range from 29.8% to 42.1% and is statistically significant (*z* = 11.42, *p* < 0.0001).

The chi‐square test and logistic regression analysis both favour the results that cattle are significantly more likely to be seropositive as compared to buffaloes when all other factors are held constant.

Data analysis showed that female animals were significantly more predominant among the studied population. Among the total animal population, just 3.72% (16/430) animals evaluated were males, whereas 96.28% (414/430) were females.

A predicted 50:50 distribution is compared to the observed sex distribution (96.28% female and 3.72% male) using the chi‐square goodness‐of‐fit test, which produces the following results: *p* value: 4.21 × 10^−^^82^, chi‐square statistic: 368.38. Strong female bias in the dataset is confirmed by this incredibly low *p* value, which shows a statistically significant deviation from a balanced male‐to‐female ratio.

Seropositivity and sex are statistically significantly associated (*p* = 0.0098). Seropositive findings are much more common in females as compared to males, which might be due to sampling bias, physiological susceptibility or exposure differences. The dataset's significant female bias is shown by the disproportion, which may be due to sampling procedure, herd composition or the demographic makeup of the studied regions. Analysing the seroprevalence findings as well as related risk factor studies needs consideration of this gender distribution.

A statistically significant relationship (*p* < 0.05) between sex and CE seropositivity *E. granulosus* was found. The binary logistic regression model indicated that males were at less risk of being seropositive as compared to females (OR = 0.093; *p* = 0.022). This shows that the probability of finding anti‐*Echinococcus* antibodies in males was almost 91% lower as compared to females after controlling for baseline risk. The finding highlights a potentially significant sex‐based differential in immunological response or exposure risk, which might be caused by behavioural, physiological or husbandry‐related factors. Furthermore, the intercept was significant (*p* = 0.001), confirming the overall fit of the model. These results show that the maintenance of hydatid infection dynamics in the research population may be more significantly influenced by female animals.

**FIGURE 4:**
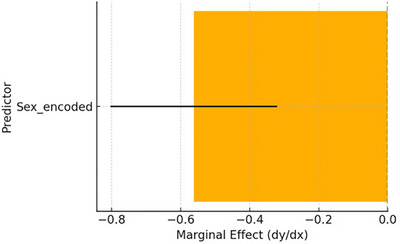
Marginal effect of sex on seropositivity based on a logistic regression model.

Figure 4 illustrates the marginal impact plot of sex on *Echinococcus* seropositivity. In line with the previous regression findings, it demonstrates that males considerably lower the expected probability of seropositivity.

The findings of the ELISA test for *E. granulosus* antibodies in animals of various age groups show significant patterns in seropositivity. The seroprevalence was comparatively low 25% (19/76) in the youngest age group (≤4 years). The age group of 4–6 years showed a more uniform distribution, with 6 positives and 5 negatives, indicating a larger prevalence of infection (54.54%; 6/11) in this smaller group.

A significant rise in seropositivity was seen in the 6–8 years group, whereas 33 animals (58.92%) were identified as positive out of 56 tested, suggesting one of the highest rates among all groups. Likewise, the 8–10 years group also showed a significant number of 61 positives out of 126 tested, representing 48.41% seropositivity. The variation between positive and negative findings reduced more in the 10–12 years group, with 42 tested positive and 40 negative, indicating almost equal distributions and a seroprevalence of 53.84%. A decrease in seropositivity was observed in the 12–14 years group category (40.42%; 19/47). At last, in the oldest age group category (>14 years), the seropositivity rate was relatively constant (47.22%; 17/36).

**FIGURE 5:**
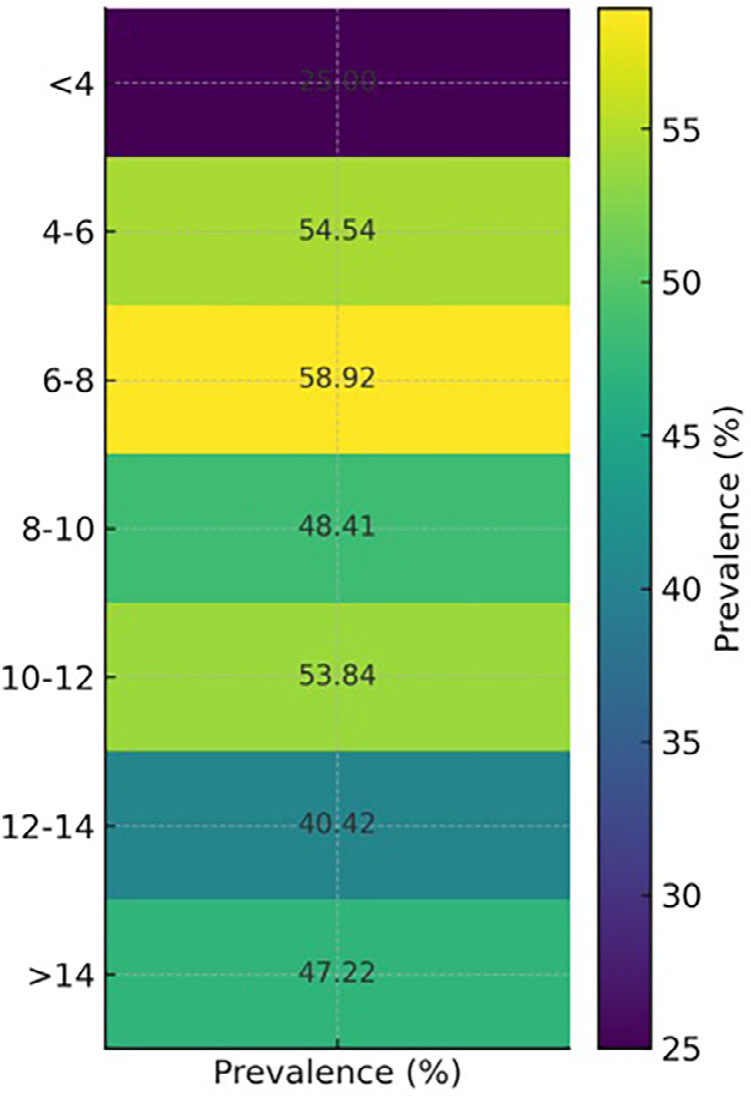
Heatmap showing the distribution of ELISA test results across different age groups.

Overall, the results show a rising trend in seropositivity from the youngest age group up to the mid‐age group categories (6–12 years), with a slight decrease in the older age groups. This trend may indicate increasing exposure over time, declining antibody levels in older animals or changing immunological response with age. In Figure 5, a heatmap showing the distribution of ELISA‐positive and negative findings among various age groups has been demonstrated.

**FIGURE 6:**
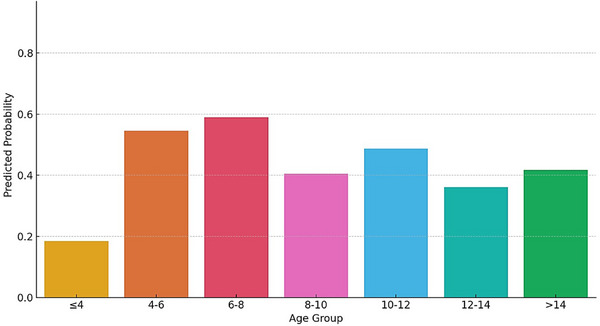
Predicted probability of seropositivity across different age groups based on logistic regression analysis.

A statistically significant relationship was found between ELISA result (positive/negative) and age group using the chi‐square test for independence (chi‐square statistic: 26.75; degrees of freedom: 6; *p* value: 0.00016). This shows a significant association between age group and seropositivity for *E. granulosus* (*p* < 0.001). Positive and negative cases are not distributed randomly among age groups. The logistic regression model estimated the probability of *E. granulosus* seropositivity by age group is displayed in a bar plot (Figure 6). The results show that the probability of seropositivity increases noticeably from the youngest group (≤4 years) to the older age group, with a notable peak observed between 6 and 12 years.

Significant variations in *E. granulosus* seropositivity rates between different age groups were found by the post hoc Tukey HSD analysis. However, the seropositivity rate was significantly higher in animals aged (≤4 years) than in older age groups, suggesting either a stronger antibody response or more exposure at a younger age. Especially, the animals in the ≤4 years group showed significantly more positivity rates than those in the 6–8 years (*p* < 0.001), 8–10 years (*p* = 0.027) and 10–12 years (*p* = 0.002) age groups. These results infer that younger animals could be more predisposed to CE. The pairwise comparisons across different age groups did not show any statistically significant difference, signifying that the youngest age group had the highest seroprevalence. This accentuates the need for early intervention and focused surveillance in the younger livestock population to minimize CE prevalence.

The results showed that there is significant variation in CE seroprevalence among animals of different breeds. The Cholistani breed showed the highest seropositivity rate (96.29%; 26/27). Comparatively, Nili‐Ravi (which comprises all buffalo samples) had the lowest seroprevalence, with only 25.25% (49/197) tested positive. A moderate level of seropositivity was shown by the Sahiwal breed, with 61.13% (118/193) testing positive. Finally, the positivity rate for crossbred animals was comparatively lower, with 30.76% (4/13) tested positive. These results indicate that the epidemiology of CE in the study population is influenced by breed‐related variations in exposure risk and immunity.

A chi‐square test was used to assess the relationship between ELISA results and breed. A highly significant relationship was found between the two variables (chi‐square = 88.86; df = 3; *p* value <0.0001). Cholistani and Sahiwal breeds showed a greater proportion of positive cases. These findings support the notion that breeds possess different immunity and are susceptible to CE differently.

The logistic regression analysis was used to determine the relationship between breed and seropositivity of *E. granulosus*. The Cholistani breed *w*as used as the reference category because of its highest documented seroprevalence. It was found that the odds of being seropositive decrease significantly for all other breeds. Especially, crossbred animals had a considerably decreased probability of testing positive (regression coefficient = −4.07; *p* = 0.0006). Likewise, the least probable group to be infected among all groups was Nili‐Ravi (buffaloes), which showed a significantly decreased probability of seropositivity, with a coefficient of −4.69 (*p* < 0.00001). Similarly, with a coefficient of −3.06 (*p* = 0.0029), the Sahiwal breed had a considerably decreased likelihood of testing positive than Cholistani. In contrast to Cholistani cattle, Sahiwal, Nili‐Ravi and crossbred animals had substantially decreased likelihood of containing *E. granulosus* antibodies, according to these negative and statistically significant coefficients. This shows a strong breed‐related impact on susceptibility or exposure risk, supporting the findings of the previous descriptive and chi‐square studies.

The association between ELISA result (positive or negative) and breed was further examined using a pairwise chi‐square. A number of statistically significant variations were found in the findings. Seropositivity rates between Sahiwal and Nili‐Ravi were significantly different (*p* < 0.0001), showing that these two breeds had very different exposures or responses to *E. granulosus*. Likewise, there was a significant difference (*p* < 0.001) between the Cholistani and Sahiwal breeds, with the Cholistani animals showing a much higher positivity rate. Cholistani animals had a higher seroprevalence than Crossbred animals, as evidenced by a substantial and statistically significant difference between the Cholistani and Crossbred breeds (*p* < 0.001). Conversely, there was no statistically significant difference in the comparisons between Sahiwal and Crossbred (*p* = 0.091) and Crossbred and Nili‐Ravi (*p* = 0.316), showing that both breed combinations had identical levels of seropositivity.

Especially, Sahiwal and Cholistani breeds showing various positivity rates, these pairwise analyses support the result that breed significantly influences the epidemiology of *E. granulosus*.

**FIGURE 7:**
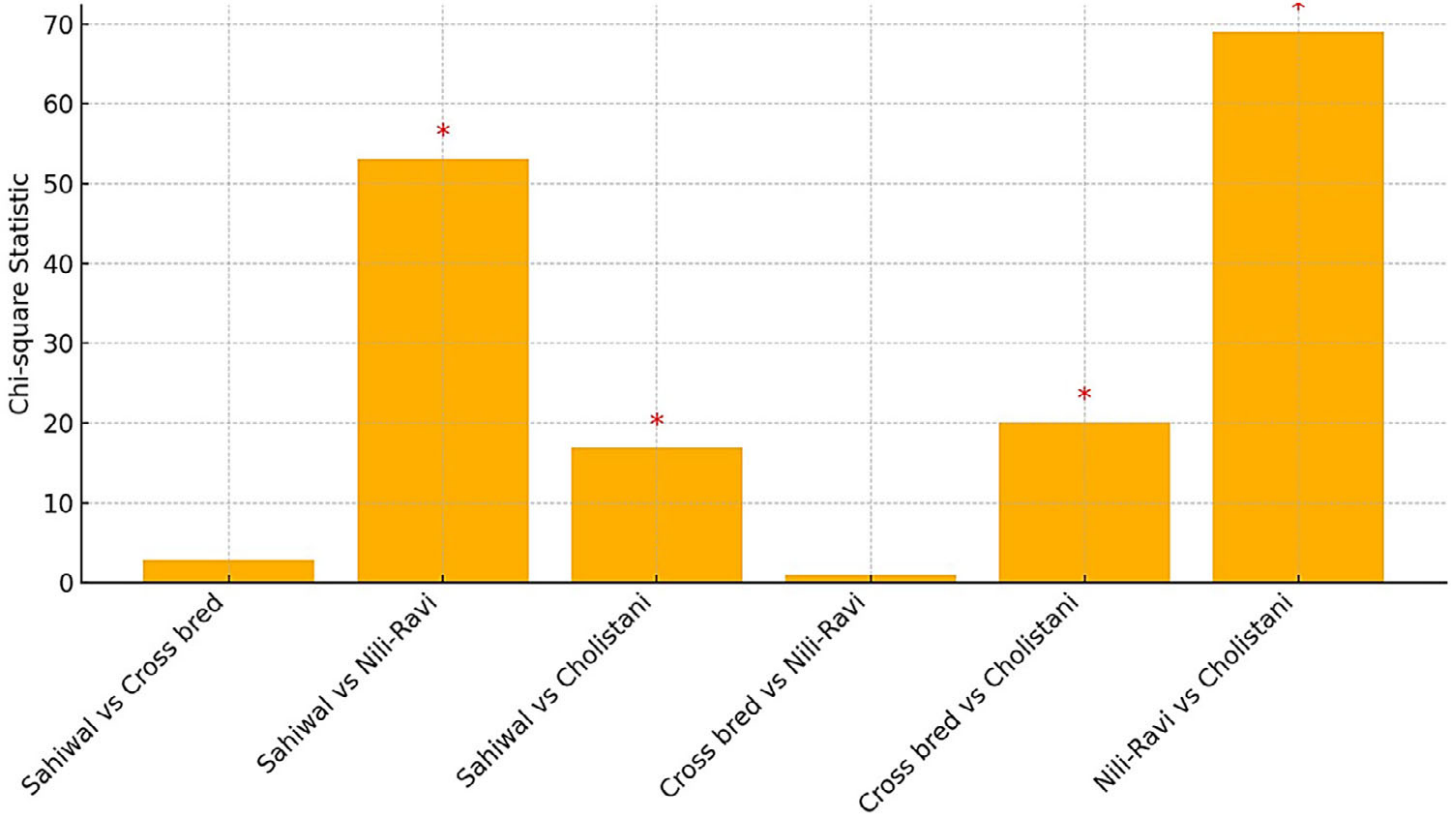
A pairwise chi‐square comparison of ELISA seropositivity results among different cattle breeds.

The chi‐square statistics for every pairwise comparison of ELISA findings by breed are shown in a bar graph (Figure 7). A statistically significant difference (*p* < 0.05) between the examined breeds is shown by an asterisk (*) above a bar.

To assess the relationship between a few chosen variables, including age, breed, sex, city of origin and seropositivity for *E. granulosus* antibodies, a multivariate logistic regression analysis was conducted. The ‘Other’ category was utilized to aggregate the unusual breed and city categories in order to improve model stability and handle sparse data concerns. The refined model, which comprised 430 complete data points, converged in seven iterations with a Pseudo *R* ^2^ value of 0.321 and a very significant likelihood ratio test (*p* < 0.0001), showing strong model fit. Male animals had significantly lower likelihoods of being seropositive than females (coefficient = −3.00; *p* = 0.0065). Age and seropositivity were positively linked, but this association was not statistically significant (*p* = 0.1916). Unreasonably large standard errors and statistically non‐significant relationships were observed for most of the breeds and cities demonstrating data scarcity or quasi‐complete isolation. The results showed sex as an important predictor of seropositivity, but the results also show the need for models to stabilize estimates for categorical variables with low sample size/representation.

Penalized logistic regression was used to solve the sparsity issue with multivariate logistic regression. When determining predictors of *E. granulosus* seropositivity, the penalized logistic regression model with L1 regularization (LASSO) offered enhanced stability and interpretability. With a macro‐averaged *F*1‐score of 0.77 and an overall model accuracy of 76.98%, the model performed well in both positive and negative classifications. Notably, for negative cases, the model had a precision of 87.56% and a recall of 71.48%, whereas for positive cases, the precision was 66.97% and the recall was 85.06%.

**FIGURE 8:**
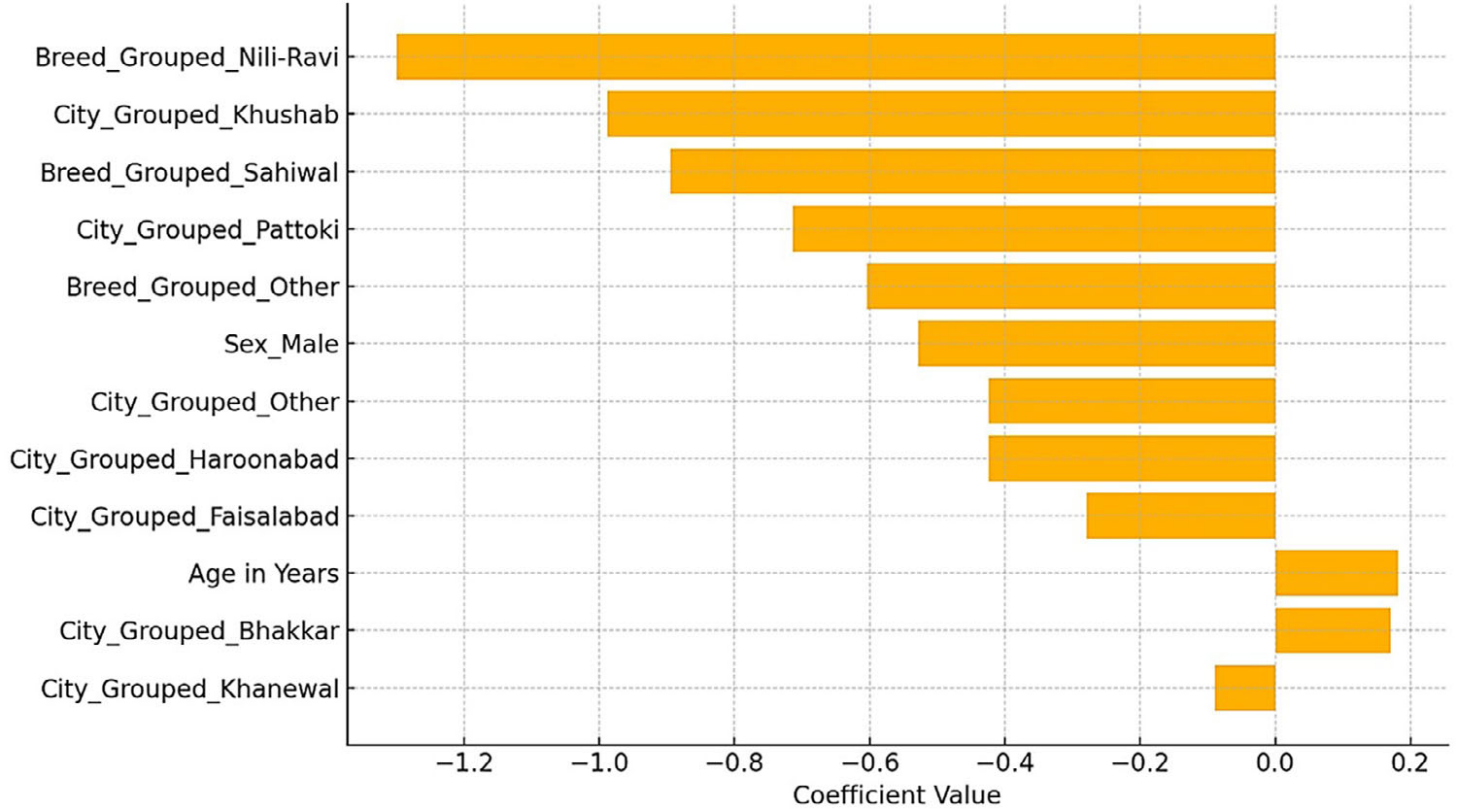
Top predictors of *Echinococcus* seropositivity identified through LASSO logistic regression.

The most significant predictors were emphasized using the LASSO model, which significantly decreased the impact of less informative variables by reducing their coefficients towards zero (Figure 8). According to LASSO logistic regression, the plot shows the most significant predictors of *E. granulosus* seropositivity. However, variables with positive coefficients have higher probabilities of testing positive, whereas those with negative coefficients have lower probabilities.

**FIGURE 9:**
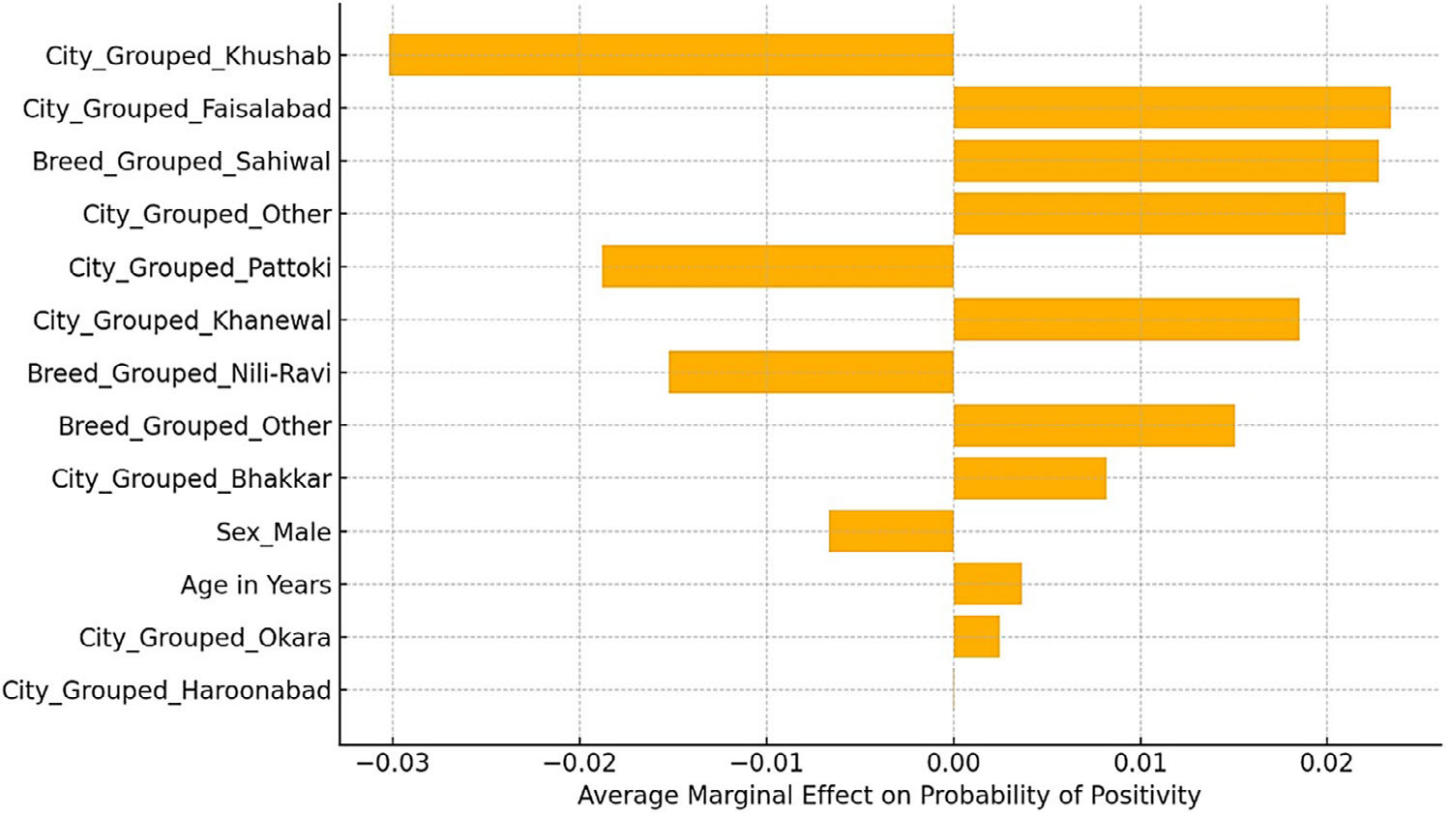
Marginal effects of predictors on the probability of *Echinococcus* seropositivity derived from a LASSO logistic regression model.

However, all other variables held constant, the marginal effects plot (Figure 9) was designed to show the average change in the expected odds of *E. granulosus* seropositivity for a single standard deviation rise in every predictor. The result is more strongly influenced by predictors having higher absolute marginal effects.

The penalized logistic regression model's marginal effects analysis offers an understanding of the ways in which specific predictors affect the odds of *E. granulosus* seropositivity. Sex was the most significant variable among those analysed, with male animals having much lower odds of testing positive than females. This reflects the outcomes of the previous logistic regression and indicates the possibility of a sex‐related exposure or susceptibility difference. Additionally, the marginal impact of age was positive; demonstrating that the odds of seropositivity rise with age may be due to long‐term exposure to parasites. The animals from Khanewal and Bhakkar exhibited a greater likelihood of seropositivity, whereas animals from Faisalabad showed a lower likelihood in comparison to the reference group. Further, the Sahiwal breed and the animals of less popular breeds were less likely to test positive, suggesting breed‐related effects. The findings of the current investigation demonstrate the crucial role of host‐related and geographic factors in finding the epidemiological landscape of *E. granulosus* infection and favour the use of penalized models for consistent and interpretable variables of choice in sero‐epidemiological research.

## 4 Discussion

Utilizing ELISA‐based testing, the current study provides an in‐depth analysis of the seroprevalence of *E. granulosus* antibodies in cattle and buffaloes across many districts in the Punjab province of Pakistan. Seropositivity varied significantly by species, geography and demographics in the overall data, suggesting significant epidemiological trends for disease control. Although a high seropositivity rate is an indicator of extensive exposure to *E. granulosus*, it cannot be regarded as active infection of the hydatid cyst. The antibodies can persist well after infection, and ELISA cannot distinguish between resolved, subclinical and active CE. Consequently, the seroprevalence in this case is more of exposure dynamics as opposed to understanding of the burden of active cysts or direct productivity losses. Active infection would be confirmed either by imaging, post‐mortem examination or molecular diagnostics.

The published assessments of commercial and recombinant‐antigen ELISAs claim sensitivity values as 70%–90% and specificity as 90%–98% (Gavidia et al. 2008; Craig et al. 2007; Siracusano et al. 2012). Our rEg‐TPx ELISA (82.3% sensitivity; 95.4% specificity) has a diagnostic performance within this acceptable range.

The foremost finding of the present investigation is significant geographical variation in the seroprevalence of *E. granulosus*. The highest seropositivity rate in Bahawalpur was 96.29%, whereas in Bhakkar and Pattoki, the seropositivity rate was 65.21% and 53.65%, respectively. On the other hand, the prevalence of 26.31% and 9.52% was significantly lower in districts like Faisalabad and Haroonabad, respectively. This geographical variation is statistically determined by the chi‐square test and logistic regression findings (*p* < 0.001), showing that geographical location is a major factor of infection risk.

Similar geographical variations were reported in the past. For example, a seroprevalence of 17.3% was reported in the Qinghai‐Tibet region of China, whereas variation of seroprevalence in Xinjiang, China has been reported between 15% and 25% (Gao et al. 2022; Zhang et al. 2015). A high seropositivity has been reported from Iranian Golestan (28.4%) and Khuzestan (35.8%) (Shafiei et al. 2021). These geographical similarities show that the dynamics of CE transmission are affected by the local animal husbandry practices, the relationship between dogs and livestock and veterinary infrastructure.

Due to the fact that echinococcosis is still a neglected disease in Pakistan, the present investigation revealed that many of the studied districts in Punjab had higher seropositivity rates than these values, underlining the crucial need for increased prevention and control measures (Alvi et al. 2020a).

The primary epidemiological finding of the current investigation shows that a high seropositivity rate of 68.24% in cattle, in comparison to buffaloes with a 19.28% rate, was observed. The logistic regression findings showed that cattle had about six times more likelihood of testing positive in comparison to buffaloes (OR = 5.87, *p* < 0.001). This variation may be caused by species‐specific variations in grazing habits, direct contact with dogs, management techniques or immune response (Dinkel et al. 2004; Sadjjadi 2006). Studies conducted in Iran revealed that cattle had greater seropositivity rates in comparison to small ruminants and buffaloes, which might be due to prolonged exposure to the environment or anatomical susceptibility (Khalkhali et al. 2018; Shafiei et al. 2021). The significant difference highlights the need for species‐specific treatments, especially in mixed herds.

Seropositivity was significantly predicted by sex, with females showing much greater rates. The logistic regression and marginal effect analyses demonstrated that sex significantly influences infection risk, despite the samples being biased towards females. Studies from Ethiopia and Kenya have also reported a similar pattern (Addy et al. 2012; Kebede et al. 2009), which may be because of variations in husbandry practices or longer productive life expectancy in females.

Another significant finding is that female animals made up the majority of the sample (96.3%), and they were more likely to be seropositive. As females are frequently kept longer for milk production, this finding may be partially the result of sampling bias, although it is statistically confirmed (OR = 0.093 for males, *p* = 0.022). Furthermore, this trend may also be influenced by females’ longer exposure times as a result of their longer lifespans. Moreover, longer exposure durations in females due to extended lifespans may also contribute to this pattern.

Age‐wise study showed that seropositivity increased from young to middle‐aged animals, peaking in the 6–8‐year (58.92%) and 10–12‐year (53.84%) age groups before slightly declining in older animals. It is interesting to note that post hoc analysis showed substantially greater seroprevalence in animals aged ≤4 years than older groups (*p* < 0.05), even though the pattern shows cumulative exposure over time. The results of assays in older animals may be affected by maternal antibody interference or early‐life infection in environments with elevated risk. Studies on livestock population in Turkey have shown comparable age‐related trends, with middle‐aged animals showing increased seropositivity (Shafiei et al. 2021).

Seropositivity was increased with age in animals from younger to middle‐aged animals (particularly in the 6–12 years age group), most probably as a result of long‐term exposure. The age‐related patterns seen in Egypt and China, where older animals had increased seropositivity, coincide with this pattern (Abo‐Aziza et al. 2019; Yang et al. 2022). It is interesting to note that a little decline in seropositivity in animals older than 12 years might be due to antibody fading or immunological senescence. The progressive rise of seropositivity among young to mid‐age categories is probably due to cumulative exposure through the years, whereas the minor age‐related decrease in the older animals may be due to a variety of biological factors, such as the depletion of antibody levels, induction of immunosenescence or selection effects where heavily infected or immunologically weakened animals might not survive to the old‐age group, leading to an apparently lower seroprevalence in the oldest group.

This study found that seropositivity varied by breed. Nili‐Ravi buffaloes (25.25%) and crossbred animals (30.76%) showed the lowest seroprevalence, whereas Cholistani cattle (96.29%), followed by Sahiwal (61.13%), had the highest rates. Cholistani cattle were used as the reference group, and logistic regression indicated that there were notable variations in infection risk among breeds. Keeping Cholistani cattle in semi‐arid pastoral systems, which are frequently marked by inadequate veterinary care and increased environmental contamination, may be the cause of their high seropositivity. Additionally, past studies have also documented breed‐related variations in parasite susceptibility, which are frequently impacted by immunological and genetic traits (Craig et al. 2007; Siracusano et al. 2012). Cholistani cattle had a higher rate, which might be caused by their semi‐nomadic grazing method, frequent contact with contaminated environments or limited access to veterinary treatment. This increased seropositivity in Cholistani cattle can be associated with breed‐selective ecological exposures, grazing regimes or genetic and immunologic characteristics and a reduced seropositivity in Nili‐Ravi buffaloes to increased resilience or different management regimes. These findings are consistent with research conducted in Saudi Arabia and Turkey, where native or indigenous breeds had an increased infection load than crossbred or commercial animals (Ibrahim 2010; Kesik et al. 2022).

Species, sex and city of origin were found to be the most important predictors of seropositivity using the multivariate logistic and penalized regression models. Variable selection was more stabilized using the LASSO model, which further increased model performance (accuracy: 76.98%). Male sex and buffalo species considerably decreased the likelihood of testing positive, based on the marginal effects plot, but cattle, specific cities (like Bhakkar) and middle‐aged animals raised the risk. In terms of CE dynamics, these results align with epidemiological models that highlight the interaction of host, environmental and geographic factors (Deplazes et al. 2017; Wen et al. 2019).

The statistical analysis showed sex and city as the most reliable predictors of seropositivity. Khanewal, Bhakkar and Bahawalpur districts were identified as high‐risk areas. This geographical variation in seropositivity may be attributed to differences in stray dog populations, slaughterhouse practices or awareness about CE spread factors often mentioned in previous spatial studies of CE (Budke et al. 2006; Craig et al. 2007).

The logistic regression model showed significant grouping and modest predictive accuracy (AUC = 0.73) about the regional difference of CE seropositivity in the study areas. Similar results have been reported from Peru and Turkey, where spatial modelling showed that social, cultural, ecological and climatic factors influence the burden of CE (Torgerson et al. 2010; Moro and Schantz 2009).

The higher rate of seroprevalence recorded in many areas suggests a significant zoonotic and economic impact, particularly considering Pakistan's large cattle (59.7 million) and buffalo (47.7 million) heads (Pakistan Economic Survey 2024). This study did not report productivity losses, organ condemnation or clinical outcomes. Previous studies have shown that subclinical CE infections can result in organ condemnation, low productivity (Budke et al. 2006); however, this study did not collect data on these outcomes. This highlights the importance of CE surveillance and control programs, including regular screening, deworming, slaughterhouse cleanliness and farmers’ awareness programs.

Some limitations of the current study include its cross‐sectional methodology, likely sampling bias and minimal representation of male animals. However, it provides useful insights into the epidemiology of *E. granulosus* in Pakistan. Our knowledge of CE transmission dynamics would be further improved by future longitudinal investigations that combine molecular diagnosis, dog deworming status and GIS‐based risk modelling. Though other factors like management system, herd size, deworming history and dog contact have been identified as some of the factors that contribute to the spread of CE, these variables were not reliable to include in our analysis. Due to sampling in both farms and slaughterhouses, the owners were often unable to verify and define the deworming habits, the size of the herd or the extent of the dog‐livestock contact. Equally, data in the management systems were not uniform and were frequently not available in case of animals sourced at slaughterhouses. It is for this reason that only variables that could be measured consistently (species, age, sex, breed and city of origin) were included in the regression analyses. Further longitudinal studies based on farm‐level studies are required to precisely measure these other risk factors.

The current study concludes by highlighting the critical need for region‐specific CE control measures in Pakistan, such as regular serological surveillance, public awareness campaigns, controlling the stray dog population and proper offal disposal. Considering one of the world's biggest livestock populations, Pakistan has to give priority to CE in its One Health program due to its documented zoonotic importance and the widespread seropositivity demonstrated in this study.

## 5 Conclusion

Using ELISA, this investigation offers the first comprehensive sero‐epidemiological picture of CE in Pakistani buffaloes and cattle. The results show a significant difference in seropositivity between species, geographical regions and breeds. In the future, molecular investigations should be carried out to have better insights into CE epidemiology in One Health perspective.

## Author Contributions

Conceptualisation: Mughees Aizaz Alvi, Majed H. Wakid, Li Li, Wan‐Zhong Jia and Muhammad Saqib. Methodology: Mughees Aizaz Alvi, Talha Javaid, Li Li, Warda Qamar, Wan‐Zhong Jia and Majed H. Wakid. Formal analysis: Mughees Aizaz Alvi, Talha Javaid, Muhammad Wasim Usmani, Warda Qamar, Junaid Amjed and Majed H. Wakid. Investigation: Muhammad Wasim Usmani, Warda Qamar, Aliza Ali, Junaid Amjed, Wan‐Zhong Jia and Muhammad Saqib. Data curation: Aliza Ali. Writing – original draft: Mughees Aizaz Alvi and Talha Javaid. Writing – review and editing: Hong‐Bin Yan and Majed H. Wakid. Supervision: Hong‐Bin Yan and Muhammad Saqib. Project administration: Hong‐Bin Yan and Muhammad Saqib. Funding acquisition: Hong‐Bin Yan and Majed H. Wakid.

## Funding

This study was supported by the Key Research and Development Program of Rikaze (RKZ2025ZD‐009), Gansu Province Joint Research Fund (24JRRA807), Innovation Program of Chinese Academy of Agricultural Sciences (CAASTIP‐2025–05, CAAS‐ASTIP‐2021‐LVRI), The Major Science and Technology Project of Gansu Province (24ZD13NA008, 23ZDNA007, 22ZD6NA001), Central Public‐Interest Scientific Institution Basal Research Fund (1610312023012) and NBCITS (CARS‐37). The APC was funded by the Deanship of Scientific Research (DSR) at King Abdulaziz University, Jeddah, Saudi Arabia. The authors, therefore, acknowledge with thanks DSR for technical and financial support.

## Ethics Statement

The authors confirm that the ethical policies of the journal, as noted on the journal's author guidelines page, have been adhered to and the appropriate ethical review committee approval has been received. The US National Research Council's guidelines for the Care and Use of Laboratory Animals were followed.

## Consent

The authors have nothing to report.

## Conflicts of Interest

The authors declare no conflicts of interest.

## Data Availability

The data that support the findings of this study are available from the corresponding author upon reasonable request.
